# Population-based analysis of the association between composite dietary antioxidant index and pediatric obesity

**DOI:** 10.3389/fnut.2025.1617384

**Published:** 2025-10-07

**Authors:** Qingnv Zhou, Yuhao Wu, Hui Xu, Hongyu Xie, Rongwei Yang, Huafei Huang

**Affiliations:** ^1^Department of Pediatrics, Jiaxing Maternity and Child Health Care Hospital, Affiliated Women and Children Hospital of Jiaxing University, Jiaxing, Zhejiang, China; ^2^Department of Neonatology, Xinhua Hospital Affiliated to School of Medicine Shanghai Jiao Tong University, Shanghai, China

**Keywords:** composite dietary antioxidant index, obesity, children and adolescents, population-based study, NHANES

## Abstract

**Objectives:**

Pediatric obesity is an increasingly serious global problem. Although much attention has been paid to the role of nutrition in pediatric obesity, no prior study has examined the association between the composite dietary antioxidant index (CDAI), the main measure of an antioxidative diet, and pediatric obesity, and this research aims to investigate this relationship.

**Methods:**

Using the National Health and Nutrition Examination Survey (NHANES) for the period 2009–2018, we examined the relationship between CDAI and pediatric obesity: body mass index (BMI) and waist-to-height ratio (WHtR) using multivariate linear regression models and smoothing fit curves. Furthermore, subgroup analyses were conducted to observe differences in these associations across various stratifying factors.

**Results:**

Our study encompassed 10,019 participants aged 6–18 years with complete data. There was a significant negative correlation between CDAI and BMI (*β* = −0.04, 95% CI: −0.09, −0.00, *p* = 0.0367) and WHtR (*β* = −0.08, 95% CI: −0.15, −0.02, *p* = 0.0089). Additionally, a one-unit increase in CDAI was linked to a 1.9% decrease in the odds of obesity as defined by WHtR (OR = 0.98, 95% CI: 0.96, 1.00, *p* = 0.0342). Notably, the negative associations between CDAI and both BMI and WHtR varied across subgroups.

**Conclusion:**

Our findings reveal a linear negative relationship between CDAI and both BMI and WHtR among American children and adolescents, offering novel insights into the potential protective role of antioxidant-rich diets against pediatric obesity.

## 1 Introduction

Pediatric obesity poses a worldwide health problem. From 1975 to 2016, there was a steady global rise in childhood and adolescent obesity rates after adjusting for age. Specifically, the obesity rate increased by 4.9% in girls and by 6.9% in boys (1). Pediatric obesity has a substantial impact on economically advanced nations. Notably, from 2017 to 2020, the obesity rate in the United States reached 19.7% (2). A 2019 report by the World Obesity Federation projects that pediatric obesity will affect 206 million individuals by 2025 (3). If not addressed in time, pediatric obesity can result in several health complications, including neurological, cardiovascular, and psychosocial problems (4). Effective weight management is crucial for ensuring normal growth and maintaining the physical and mental well-being of children and adolescents.

Healthy eating habits constitute an effective strategy for promoting weight loss in children and adolescents (5). In recent years, studies have investigated associations between various dietary factors and pediatric obesity, including caffeine intake, flavanone intake, and adherence to the Mediterranean diet pattern, among others (6–8). Studies have highlighted the significant role of dietary antioxidants in the prevention and treatment of obesity (9). The composite dietary antioxidant index (CDAI) (10) is considered one of the most reliable and effective indicators for the assessment of an individual’s antioxidant intake. So far, however, no research has investigated whether CDAI is associated with pediatric obesity in the United States.

Body mass index (BMI) is a widely used obesity indicator in epidemiological studies and clinical settings due to its simplicity, practicality, and ease of use. However, it does not differentiate between different types of body fat and is not accurate or reliable as a proxy for fat mass, which may restrict its use in certain studies (11). Central obesity, also known as abdominal obesity, has increasingly prominent detrimental effects on various metabolic diseases compared to generalized obesity (12). The waist-to-height ratio (WHtR) is a validated indicator of abdominal fat accumulation and provides an accurate measure of central obesity. Studies have demonstrated that WHtR outperforms other conventional anthropometric measures in assessing obesity among adults, children, and adolescents (13). Consequently, the present study aims to investigate the relationship between CDAI and pediatric obesity, as assessed by BMI and WHtR.

## 2 Materials and methods

### 2.1 Selection of participating populations

This study exclusively utilized data from the National Health and Nutrition Examination Survey (NHANES) database, a nationally representative resource created by the National Center for Health Statistics. The database uses advanced probabilistic sampling methodologies and diverse approaches for assessing the nutritional status and health of Americans. It contains a wide array of information, encompassing demographic details, dietary patterns, health examinations, laboratory findings, and surveys.

**Figure 1:**
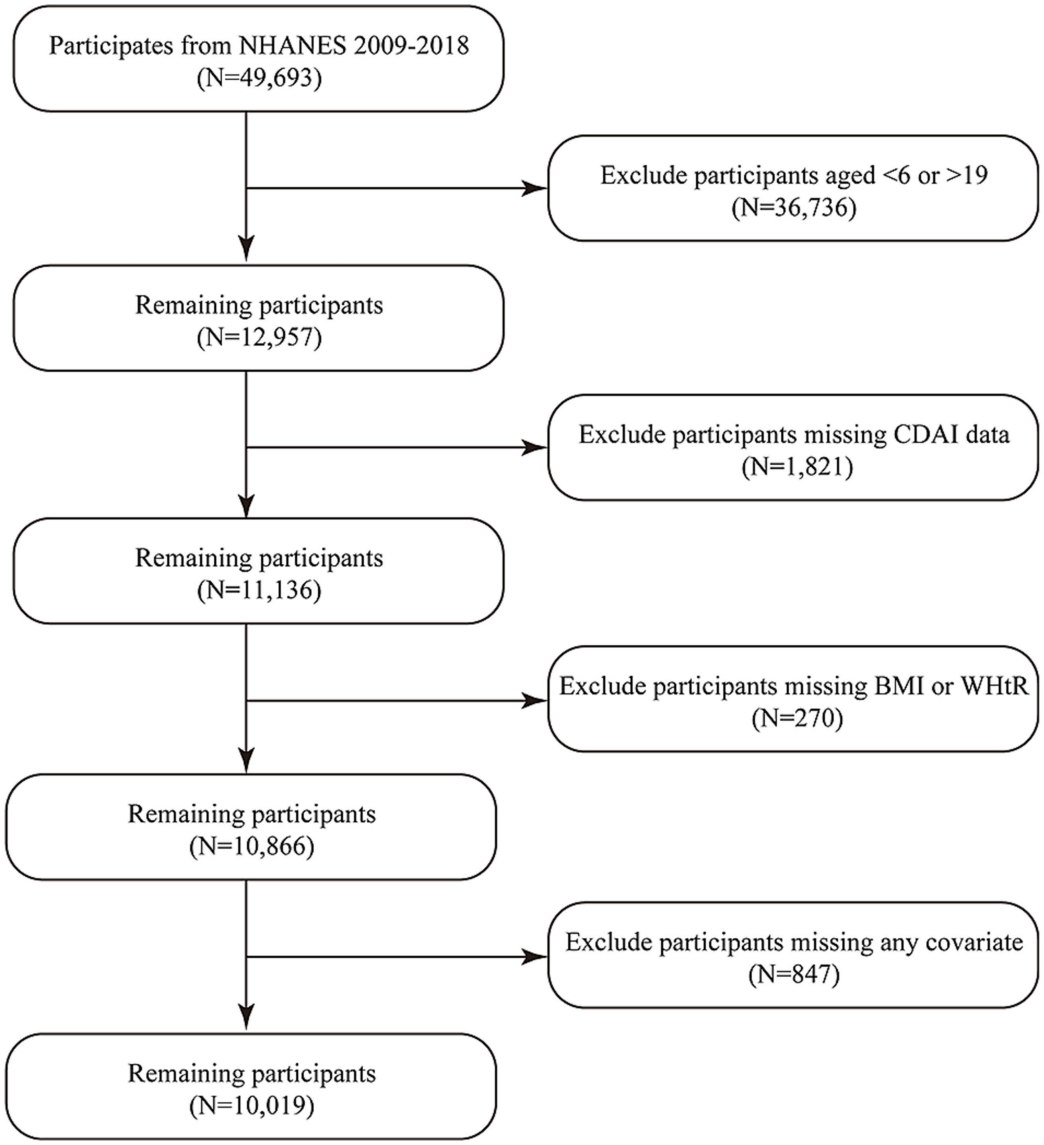
Process of participants’ inclusion.

This study initially included data from all participants in the NHANES database spanning 10 years from 2009 to 2018. From the initial pool of 49,693 participants, we excluded those aged outside the 6–18 range (*N* = 36,736), as well as those with missing data (*N* = 2,938), among which 1,821 lacked CDAI data, 270 lacked BMI or WHtR data, and 847 lacked data for any of the covariates. Ultimately, 10,019 pediatric and adolescent participants were included in the study. Figure 1 illustrates the specific participant selection process in detail.

### 2.2 Exposure variable: CDAI

The data utilized for calculating the CDAI were derived from the intake of six antioxidants recorded during two separate 24-h food recall interviews in the NHANES database, with the final results considered as the midpoint between the two measurements. The six antioxidants in question are carotenoids (including *α*-carotene, *β*-carotene, β-cryptoxanthin, lycopene, lutein, and zeaxanthin), along with vitamin A, vitamin C, vitamin E, zinc, and selenium. It is important to recognize that the CDAI values exclude dosages of antioxidants obtained through dietary supplements or pharmaceuticals.

The CDAI values were computed using an adapted version created by Wright et al. (10). The standardization process involved three sequential steps: first, the mean consumption value for each antioxidant was deducted from its corresponding 2-day mean intake; next, the data were normalized by dividing each value by the population’s standard deviation; and finally, the standardized values for all antioxidants were summed (14). In other words, the CDAI is the cumulative result of each antioxidant being standardized before summation.

### 2.3 Outcome variables: BMI and WHtR

The data utilized for calculating BMI were derived from anthropometric measurements in the NHANES database, collected by trained health professionals at mobile examination centers. BMI is found by dividing weight by the square of height, in units of kg/m^2^. A higher BMI value indicates a higher degree of generalized obesity. Apart from continuous BMI data, we also used these data to evaluate the prevalence of childhood and adolescent obesity. Obesity in children between 2 and 19 years is categorized by the Centers for Disease Control and Prevention (CDC) using BMI growth charts (15), classifying those with a BMI at or above the 95th percentile as obese. Notably, due to the characteristics of the growth charts, the cutoff values vary across different ages and genders.

Similarly, the data utilized for calculating the WHtR also originate from anthropometric measurements in the NHANES database. The WHtR is determined by dividing waist size by height, using the same measurement units for both, and is expressed as a percentage (%). A higher WHtR value indicates a higher degree of central obesity. Central obesity, as assessed by WHtR, is defined as a WHtR of 50% or greater (16).

### 2.4 Covariates

Based on previous related studies (17–19), we selected seven variables that may potentially influence the association between CDAI and pediatric obesity as covariates to analyze potential confounding factors. These seven variables are: gender, age, ethnicity, poverty–income ratio (PIR), protein intake (g), carbohydrate intake (g), and fat intake (g).

The representation of gender is in the form of boys or girls, while racial categories include Mexican American, other Hispanic, Non-Hispanic White, Non-Hispanic Black, and other ethnicities. Additionally, for statistical analysis and convenience, continuous variables are grouped as follows: age is classified into children (6–8 years) and adolescents (9–18 years); PIR is categorized as low (≤1.3), medium (>1.3, ≤3.5), and high (>3.5); and protein, carbohydrate, and fat intakes are each divided into tertiles, denoted as T1, T2, and T3, respectively, in ascending order.

### 2.5 Statistical analysis

The exposure variable in this study was the value of CDAI, while the outcome variables were BMI and WHtR. Multivariable logistic regression analysis was used to investigate the relationship between the exposure variable and the outcome variables. Three models were constructed by adjusting for different covariates. In particular, no adjustment was made for any covariates in the crude model. Model 1 made a minimal adjustment for sex, age, and ethnicity. Model 2, a fully adjusted model, adjusted for all covariates, including sex, age, ethnicity, PIR, protein intake, carbohydrate intake, and fat intake. The correlation was measured by calculating the corresponding *β* coefficients, the odds ratio (OR), and the 95% confidence interval (CI).

In this study, we used the t-test to analyze continuous data and the chi-square test to show categorical data as percentages. A *p*-value less than 0.05 meant the results were statistically significant. All relevant statistical tests and methods were conducted using R-Software version 4.0.

## 3 Results

### 3.1 Baseline characteristics of participants

The research encompassed 10,019 participants, both adolescents and children, aged between 6 and 18 years. Table 1 shows an in-depth overview of the initial attributes of the participants, categorized according to age. Adolescents had higher CDAI values compared to children (0.09 ± 4.18 vs. -0.29 ± 3.07), exhibited higher BMI values (22.96 ± 6.14 vs. 17.58 ± 3.58) and WHtR values (49.49 ± 8.57 vs. 47.94 ± 6.30), and were more likely to be classified as having general obesity (21.64% vs. 19.13%) and central obesity (38.30% vs. 27.36%).

**Table 1:** Characteristics of the study population stratified by age groups.

Variable	Overall (*N* = 10,019)	Children (*N* = 2,394)	Adolescents (N = 7,625)	*p*-value
Age (Mean ± SD)	12.12 ± 3.99	7.00 ± 0.82	13.73 ± 3.13	<0.001
Gender (%)				0.145
Boys	5,101 (50.91)	1,250 (52.21)	3,851 (50.50)	
Girls	4,918 (49.09)	1,144 (47.79)	3,774 (49.50)	
Ethnicity (%)				0.883
Mexican American	2,186 (21.82)	520 (21.72)	1,666 (21.85)	
Other Hispanic	1,029 (10.27)	254 (10.61)	775 (10.16)	
Non-Hispanic White	2,949 (29.43)	716 (29.91)	2,233 (29.29)	
Non-Hispanic Black	2,427 (24.22)	574 (23.98)	1,853 (24.30)	
Other	1,428 (14.25)	330 (13.78)	1,098 (14.40)	
PIR (Mean ± SD)	2.03 ± 1.54	1.96 ± 1.52	2.05 ± 1.54	0.012
Protein Intake, gm/day (Mean ± SD)	71.22 ± 30.97	64.53 ± 22.56	73.32 ± 32.90	<0.001
Carbohydrate Intake, gm/day (Mean ± SD)	253.27 ± 95.34	243.97 ± 75.16	256.19 ± 100.67	<0.001
Fat Intake, gm/day (Mean ± SD)	73.98 ± 33.13	68.47 ± 26.21	75.71 ± 34.84	<0.001
CDAI	−0.00 ± 3.94	−0.29 ± 3.07	0.09 ± 4.18	<0.001
BMI, kg/m^2^	21.67 ± 6.08	17.58 ± 3.58	22.96 ± 6.14	<0.001
Generalized obesity assessed by BMI				0.009
No	7,911 (78.96%)	1,936 (80.87%)	5,975 (78.36%)	
Yes	2,108 (21.04%)	458 (19.13%)	1,650 (21.64%)	
WHtR (%)	49.12 ± 8.11	47.94 ± 6.30	49.49 ± 8.57	<0.001
Central obesity assessed by WHtR				<0.001
No	6,444 (64.32%)	1,739 (72.64%)	4,705 (61.70%)	
Yes	3,575 (35.68%)	655 (27.36%)	2,920 (38.30%)	

PIR, Poverty–income ratio; CDAI, Composite dietary antioxidant index; BMI, Body mass index; WHtR, Waist-to-height ratio.

### 3.2 Associations of CDAI with BMI and WHtR

Table 2 displays the outcomes of a multivariate logistic regression study examining the link between CDAI and BMI, along with CDAI and WHtR. There was an inverse relationship between CDAI and BMI (*β* = − 0.05, 95% confidence interval: −0.08, −0.02) as well as WHtR (*β* = −0.18, 95% CI: −0.22, −0.14). After adjustment for gender, age, and ethnicity (Model 1), there was still a significant correlation between CDAI and BMI, as well as between CDAI and WHtR, with *β* values between −0.08 (95% CI, −0.11, −0.05) and −0.17 (95% CI, −0.21, −0.13). Similarly, CDAI had a negative association with BMI (*β* = −0.04, 95% CI: −0.09, −0.00, *p* = 0.0367) and WHtR (*β* = −0.08, 95% CI: −0.15, −0.02, *p* = 0.0089). These findings suggest that for every additional unit of CDAI, BMI decreases by approximately 4%, while WHtR decreases by 8%.

**Table 2:** Association of CDAI and BMI, WHtR.

Variables	Crude model	Model 1	Model 2
	β (95% CI) *p*-value	β (95% CI) *p*-value	β (95% CI) *p*-value
BMI	−0.05 (−0.08, −0.02) 0.0013	−0.08 (−0.11, −0.05) < 0.0001	−0.04 (−0.09, −0.00) 0.0367
WHtR	−0.18 (−0.22, −0.14) < 0.0001	−0.17 (−0.21, −0.13) < 0.0001	−0.08 (−0.15, −0.02) 0.0089

CDAI, Composite dietary antioxidant index; BMI, Body mass index; WHtR, Waist-to-height ratio. The crude model did not adjust for any covariates; Model 1 adjusted for gender, age, and ethnicity, and Model 2 further adjusted for PIR, protein intake, carbohydrate intake, and fat intake based on Model 1.

Furthermore, we represented BMI and WHtR as categorical variables indicating the presence or absence of generalized obesity and central obesity, respectively, to further examine the association between CDAI and pediatric obesity, with the results presented in Table 3. Both the crude model and Model 1 similarly demonstrated significant negative correlations between CDAI and generalized obesity, as well as between CDAI and central obesity. After adjusting for all covariates, the odds of being classified as central obese according to WHtR decreased by 1.9% for each additional unit of CDAI (OR 95% CI: 0.963, 0.999, *p* = 0.03419). Interestingly, Model 2 showed no significant association between CDAI and BMI-defined generalized obesity (OR = 0.982, 95% CI: 0.962, 1.003, *p* = 0.09812).

**Table 3:** Association between CDAI and obesity as measured by BMI and WHtR.

Variables	Crude model	Model 1	Model 2
	OR (95% CI) *p*-value	OR (95% CI) *p*-value	OR (95% CI) *p*-value
Generalized obesity	0.972 (0.959, 0.984) 0.00002	0.970 (0.957, 0.983) < 0.00001	0.982 (0.962, 1.003) 0.09812
Central obesity	0.960 (0.949, 0.970) < 0.00001	0.962 (0.951, 0.973) < 0.00001	0.981 (0.963, 0.999) 0.03419

CDAI, Composite dietary antioxidant index; BMI, Body mass index; WHtR, Waist-to-height ratio. The crude model did not adjust for any covariates, Model 1 adjusted for gender, age, and ethnicity, and Model 2 further adjusted for PIR, protein intake, carbohydrate intake, and fat intake based on Model 1.

### 3.3 Smooth curve fitting

**Figure 2:**
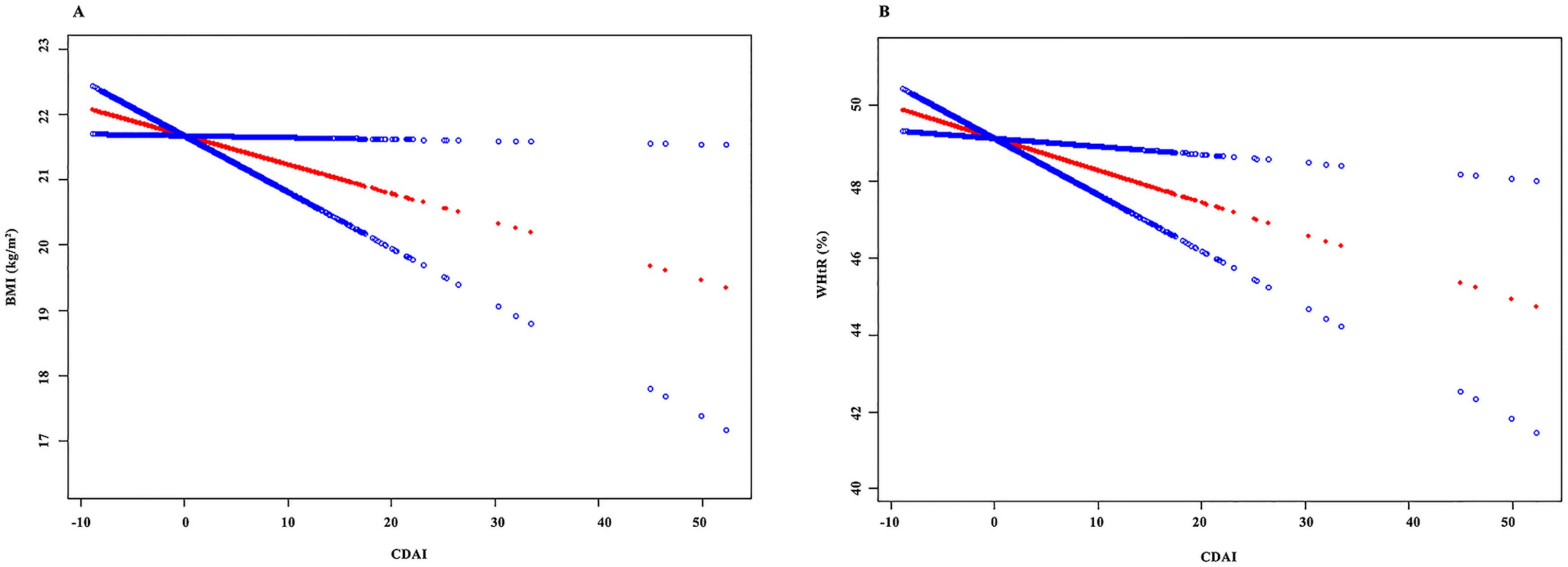
Results of the smooth curve fitting. **(A)** CDAI and BMI; **(B)** CDAI and WHtR.

The results of the smooth curve fitting are shown in Figure 2, indicating that no non-linear relationships were detected between CDAI and either BMI or WHtR.

### 3.4 Subgroup analysis

**Figure 3:**
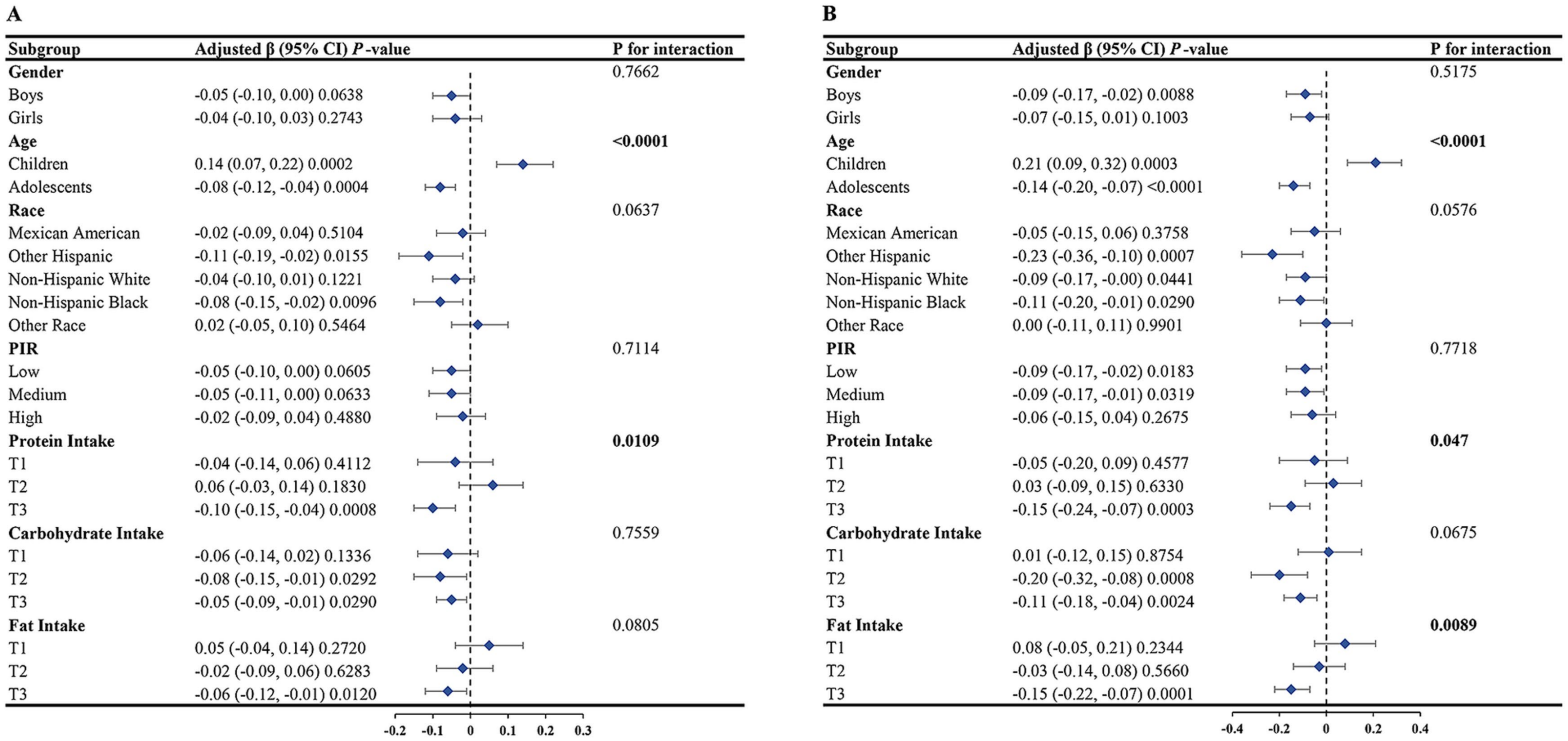
Results of the subgroup analysis. **(A)** CDAI and BMI; **(B)** CDAI and WHtR.

Our analysis of subgroups was segmented according to multiple criteria, including gender, age, ethnic background, PIR, protein intake, carbohydrate intake, and fat intake. The results, shown in Figure 3, show that the relationships vary.

Notably, we found statistically significant interaction effects (*p* < 0.05) for the relationship between CDAI and BMI within subgroups defined by age and protein consumption, but not within other subgroups. The inverse relationship between the two was more evident in adolescents and in individuals with higher protein consumption.

The relationship between CDAI and WHtR demonstrated statistically significant interaction effects across subgroups defined by age, protein consumption, and fat consumption (*p* < 0.05), whereas it was insignificant in other subgroups.

## 4 Discussion

In our research, we investigated the relationships among CDAI, BMI, and WHtR in US youth aged 6–18. After taking into account all the relevant variables, we found that CDAI was associated with both BMI and WHtR significantly, indicating a trend where higher CDAI values correlate with decreased BMI and WHtR levels. This implies that boosting antioxidant consumption in daily meals could aid in combating and handling pediatric obesity.

To the best of our understanding, the present analysis marks an initial attempt to assess the linkage between CDAI and pediatric obesity in the US. Despite the scarcity of research on this connection, similar discoveries can be traced back to prior studies. Key research areas focusing on the relationship between antioxidant-rich diets and obesity predominantly concentrate on adults: a study involving US adults (20) exhibited an inverse relationship between CDAI and areas of visceral fat, hinting that an elevated CDAI might ameliorate visceral obesity. Another study targeting elderly Americans (21) unveiled an L-shaped inverse relationship between CDAI and sarcopenic obesity. Furthermore, a prospective study involving middle-aged and elderly individuals in Rotterdam (22) suggested that dietary antioxidant intake could favorably influence lean body mass and overall body composition in this demographic. These conclusions broadly align with our findings. Research on children and adolescents is scarce, with just one comparable study (23) identified: a cross-sectional examination among Greek elementary school students aged 10–12 exploring the connection between the dietary antioxidant index and BMI. When BMI was considered a continuous measure, a negative association was noted, mirroring our results. However, when BMI was categorized to evaluate obesity, that study still demonstrated a significant negative correlation, which was not apparent in our study. Variations in research results could stem from disparities in the size of the sample, age demographics, and geographical locations.

It is worth mentioning that in our study, after adjusting for all confounding factors, the association between CDAI and generalized obesity (measured by body mass index, BMI) was not statistically significant (*p* > 0.05). In contrast, a significant inverse association (*p* < 0.05) persisted between CDAI and central obesity (measured by waist-to-height ratio, WHtR). This finding highlights a notable divergence in the relationship between CDAI and different types of obesity. In previous research, we have also noted variations in the associations between BMI and WHtR with different health conditions. A comprehensive analysis conducted by Ashwell et al. (24) revealed that WHtR is more effective than BMI in identifying cardiometabolic risk. Conversely, a meta-analysis by Lo et al. (25) indicated that BMI is more accurate than WHtR in screening for hypertension.

Although the exact mechanisms by which the CDAI is associated with pediatric obesity remain incompletely understood, they may be related to the biochemical mechanisms between oxidative stress and obesity. Biochemical imbalance, characterized by an imbalance between antioxidants and free radicals, serves as a pivotal aspect in the development of obesity and its associated issues (26). Diet holds a fundamental position in regulating blood redox status and affording protection against oxidative and nitrosative agents (27). Research has shown that diets abundant in antioxidant substances can safeguard living beings from biochemical imbalances (28). The CDAI offers a thorough assessment of total antioxidant consumption derived from food, incorporating six components that play vital parts in mitigating stress-triggered oxidant alterations (29). It has been reported that vitamin A serves as an important regulator of adipose tissue development (26). Vitamin C exhibits antioxidant and protective effects against oxidative stress and obesity (30). Vitamin E can diminish fat tissue scarring, irritation, and oxidative damage, thereby enhancing the physiological condition of obese patients (31). The double-bond system endows carotenoids with antioxidant properties, and carotenoids and their metabolites trigger anti-obesity activities through multiple mechanisms (32). Zinc and selenium play vital roles in safely combating inflammation and maintaining redox balance (33). Overall, the CDAI can be used to comprehensively assess dietary antioxidant intake.

Subgroup analysis revealed significant differences in the associations between CDAI and BMI, as well as between CDAI and WHtR, across age groups. This may be attributed to factors such as growth and development characteristics, nutritional needs, and dietary habits of children and adolescents at different age stages. For instance, during childhood, due to rapid growth and development and high nutritional demands (34), the intake of antioxidants in the diet may have a more pronounced impact on obesity and growth and development. In contrast, during adolescence, with differences in growth and development characteristics, dietary variations, and changes in lifestyle (35, 36), the relationship between dietary antioxidant intake and obesity may undergo alterations. Similarly, protein intake also modifies the relationship between CDAI and obesity. Protein is a vital macronutrient needed for growth and maturation, indispensable for maintaining the health of muscles, bones, the immune system, and more (37). Variations in protein intake may influence weight gain, body fat distribution, and metabolic status among children and adolescents (38). One study suggests that the effect of high protein intake on obesity varies by age (39). In particular, we found that CDAI and WHtR were significantly different among fat intake subgroups (*p* < 0.05). According to one study, high-fat intake increases oxidative stress levels (40), which may be more evident in terms of abdominal manifestations.

The research presents multiple advantages. Initially, it uses a substantial sample that represents the entire nation. Secondly, the comprehensive assessment of obesity through BMI and WHtR facilitates the observation of differences between these two indicators. Additionally, a variety of models were utilized to account for numerous possible confounding elements, and analyses of subgroups were performed to bolster the solidity of our results. Nonetheless, acknowledging the constraints of our study is crucial. Our capacity to deduce causal links is constrained by the study’s cross-sectional nature. Although many covariates are included, other factors affect the results. Finally, the potential for confounding bias and reporting bias remains.

## 5 Conclusion

The findings of our research reveal a direct inverse relationship between CDAI and both BMI and WHtR in American youths and adolescents. Consuming a diet high in antioxidants could be beneficial in preventing and alleviating pediatric obesity.

## Data Availability

Publicly available datasets were analyzed in this study. This data can be found at: https://wwwn.cdc.gov/nchs/nhanes/default.aspx.
